# Executive function in high-functioning autism: Decision-making consistency as a characteristic gambling behaviour

**DOI:** 10.1016/j.cortex.2018.01.013

**Published:** 2018-10

**Authors:** Hsuan-Chen Wu, Sarah White, Geraint Rees, Paul W. Burgess

**Affiliations:** a Institute of Cognitive Neuroscience, University College London, UK; b Wellcome Trust Centre for Neuroimaging, University College London, UK

**Keywords:** Executive function, Autism, Gambling, Repetitive behaviour

## Abstract

Restricted and repetitive patterns of behaviours, interests, or activities are a critical diagnostic criterion for autism spectrum disorder (ASD). Previous studies using gambling paradigms with ASD populations have identified that, unlike typically developed control participants, people with a diagnosis of ASD tend to maintain particular response patterns regardless of the magnitude of potential outcomes to uncertain gains or losses. Here we designed a gambling test that permitted calculation of the response consistency in gambling choices in situations that presented varying expected outcomes in terms of gains or losses. The task was administered to 33 adults with a diagnosis of ASDs and compared to a group of 47 typically-developed (TD) control participants who were matched for age and IQ. When presented with choices where participants could either make a risky gamble or a safe choice in terms of gains or losses (e.g., 20% chance of winning £5 *vs.* 100% chance of winning £1), the ASD participants did not differ from the TDs in their overall risk-taking behaviour. However, they were more consistent in their individual choices from trial to trial. Furthermore, the proportion of participants who either implemented an invariate response strategy (e.g., either always choosing the most risky or most “safe” option) was significantly higher in the ASD group compared with the controls. Additionally, while the ASD group were slower to make their responses in the win frame and the first half of the lose frame, by the end of the task their decision times were the same as the TD controls. These findings suggest that the ASD tendency towards repetitive behaviour may demonstrate itself even in high-level decision-making tasks, which needs to be understood if we are to be sure what such tasks are measuring.

## 1 Background

Rumiati and Humphreys (2015) highlight the extraordinarily rapid recent development of the field of “social cognitive neuroscience”, and describe this field as facilitating “the development of models attempting to bridge social cognition with neuroscience”. One of the ways in which this is being conducted is to apply the methods developed from cognitive neuroscience and neuropsychology to the study of people with autism, particularly those related to “executive dysfunction” (e.g., Spitzer, White, Mandy, & Burgess, 2016). However, the possibility exists that the individual differences in performance on a particular paradigm that exist in people with autism do not share a common basis with typically-developed people, or those with acquired brain damage. This would challenge the drawing of inferences from these findings. For instance, White (2013) argues that performance on tests of executive function may not reflect a true “executive dysfunction”, but instead the failure to form an implicit understanding of what the experimenter expects from the participant in performance of the task, leading to odd and idiosyncratic behaviour. It is argued that in these situations, the source of the impairment is actually one of mentalizing or some other social impairment in social cognition rather than of non-social executive function. This is a particularly critical issue, since impairments on tests purportedly of executive function are prevalent in autism (Hill, 2004).

Indeed, there is much debate about the significance of findings on executive function tests in terms of understanding the features of autism (Leung et al., 2016, Ozonoff, 1997). Some have argued that the dysexecutive features of autism are primary to the condition (e.g., Russell, 1997), and Pellicano (2007) has argued that executive function may be a necessary precursor to development of theory of mind (see also Ozonoff & McEvoy, 1994). But others have suggested more specific or complex relations between the various features of social cognition (including mentalizing and theory of mind), repetitive behaviour, and “executive dysfunction” (by which is usually meant problems with dealing with novel situations, monitoring and adjusting behaviour, inhibition, initiation etc.). For instance, while several authors have noticed a correlation between executive function problems and impairments in social and communication in autism (e.g., Gilotty, Kenworthy, Sirian, Black, & Wagner, 2002), others have noted a relationship between executive function problems and repetitive behaviours but not sensory features (Boyd, McBee, Holtzclaw, Baranek, & Bodfish, 2009). Others maintain that there may be a relation between repetitive behaviour and only some measures of executive functioning (South, Ozonoff, & Mcmahon, 2007). So there is a general contrast between those who see executive dysfunction as core and probably causal to the presentation of behavioural and social features of autism (e.g., repetitive behaviour, mentalizing), and those who suggest that the relations between these constructs might be more complex.

This latter view would be easy to justify from what we now know about the functions of the frontal lobes and their supporting structures within the brain. Indeed, given what we know about the location of structures within the prefrontal cortex that support social competencies like mentalising and theory of mind, and also various executive functions, it is a possibility that any relation in performance is in effect merely epiphenomena. In neurological patients with acquired damage, there is no “executive (or “frontal lobe”) syndrome”: the various dysexecutive features show a high degree of dissociation (SeeBurgess and Stuss, 2017 for review), with, seemingly, each function having its own neuroanatomical substrates.

For instance, multiple studies of mentalising and social cognition both in neurological patients and also neuroimaging of healthy brains have isolated medial PFC, including caudal medial areas 10 and 11 (frontopolar and orbitofrontal regions) as being a critical part of the brain network that supports social cognition and theory of mind (e.g., Blair and Cipolotti, 2000, Gilbert, Spengler, Simons, Steele et al., 2006, Shammi and Stuss, 1999). This region is extremely close to that which supports executive functions such as multitasking, prospective memory, and task initiation speed (e.g., Burgess et al., 2000, Burgess et al., 2001, Burgess and Wu, 2013; Volle, Gonen-Yaacovi, de Lacy Costello, Gilbert, & Burgess, 2011). So any developmental or acquired condition that might affect this general region might cause a regular co-occurrence in problems with theory of mind and some executive abilities merely because the anatomical substrates are close together in the brain rather than that the processing is shared or that there is a causal link between them. This may be one explanation for the high frequency of impairments in e.g., multitasking and also theory of mind in people with autism (e.g., White, Burgess, & Hill, 2009). A more complex but related possibility is that executive and social difficulties might be secondary to poor functional connectivity within the brain (Just, Cherkassky, Keller, Kana, & Minshew, 2007). In this case the process that has caused the poor connectivity may just be a mediator variable. But in neither case need there be a direct link at an information processing level between the social or behavioural problems and the executive ones.

Part of the difficulty in attempting to disentangle these various factors and influences is that most, if not all, of the studies that have examined the relation between them have been correlational in design. Typically, measures of social cognition and a separate measure (either psychometric or rating scale) of executive function are administered, and the correlation between them is examined. But these measurements are rarely likely to be independent. Not only might problems with implicit understanding of what is expected of the participant affect what they do on an executive function task (as White's triple-I hypothesis contends), but also behavioural features such as a repetitive tendency might in theory determine behaviour on an executive function task, contributing to variance in performance independently from variance in the “executive function construct (e.g., inhibition, decision-making or whatever) that is the intended focus of measurement of the task.

In these ways the investigation of the relation between social and executive deficits in autism mirrors that which has been conducted in neurological patients with acquired deficits over the last 50 years in particular. For instance, it has long been known that lesions induced through psychosurgery cause mood changes, as well as changes in social behaviour and also poor performance on executive function tasks even in the context of preserved IQ (Intelligence Quotients) (Bridges et al., 1994, Kartsounis et al., 1991). One such highly influential line of enquiry started approximately 30 years ago with Eslinger and Damasio's (1985) case EVR. This person, who had suffered damage to the orbital and inferior medial frontal cortices, showed a profound change in social behaviour, reminiscent of Harlow's famous case Phineas Gage. He was previously a successful accountant, and his current intellectual and memory abilities were above average. He also performed many neuropsychological tests very well. But following his frontal lobe damage he was involved in two divorces in two years and drifted unsuccessfully through several jobs. He got involved in a risky business venture with a person of questionable repute, and went bankrupt, and had great problems making decisions. For instance deciding on a restaurant to go to could take 2 h or more. Bechara and Damasio (2005) describe a root cause of the problems of patients like EVR as being that “the choices they make are no longer advantageous—the patients often decide against their best interests—and are remarkably different from the kinds of choices they were known to make in the pre-morbid period”. The idea was that the frontal lobe damage had rendered the patients relatively insensitive to reward, and that decision-making is influenced by signals that have their origin in bioregulatory processes (sometimes unconscious) related to emotions. It was thus an attempt to explain the complex relationship between emotion, social behaviour, and conscious decision-making. This “somatic marker hypothesis” was tested using a gambling procedure called the Iowa Gambling Task (discussed below). A key advance of this line of enquiry was in seeing disorders of social behaviour and economic decision-making as sharing a common cause.

If altered reward processing as indexed by performance on gambling tasks might be one cause of unusual social behaviour in these patients, then it seems reasonable to ask whether other populations who show unusual social behaviour (e.g., ASD) might also show unusual behaviour on gambling tasks. Accordingly, in this study we look at performance an ASD population on this class of economic decision-making task that was first created in the context of a model that presupposed a transparency between symptoms of social impairment (in patient EVR), and decision-making (the Iowa Gambling Test or IGT; Bechara, Damasio, Damasio, & Anderson, 1994). If altered reward or risky decision-making sensitivity might be one reason for at least some of the differences in social behaviour between ASD and typically developed (TD) populations, then one might expect the ASD group to behave very differently than the TDs on a task like the Iowa Gambling Task in terms of the risks they are willing to take.

However, if this is not a good account of social behaviour oddities in ASD, then we might expect baseline risk-taking behaviour in ASD to be similar to that found in TD populations. By contrast, if explanations such as impaired mentalizing (through an inability to determine implicit social rules) might lead to unusual risk decision-making, then one might perhaps expect the ASD population to be less constrained – or variable – in their choices than the TDs. However, as outlined above, one other important feature of autism that might also be relevant to the choices ASD populations make in risk decision-making could be the tendency towards restricted, repetitive, and stereotyped behaviours, which is one of the core symptoms of autism spectrum disorder (ASD), along with social and communication difficulties.

Repetitive mannerisms are characterised by behaviours that are high in frequency, invariant in manner, and are associated with a desire for sameness in the environment (Kanner, 1943). It also includes a cognitive component, in the form of preoccupation with restricted interests which often occur alongside non-functional routines or rituals (Diagnostic and Statistical Manual of Mental Disorders 5th ed. (2013). These thoughts have been characterised as ‘desire for sameness to a marked degree’ (Prior & Macmillan, 1973), mimicking the desire for sameness in the environment. Turner, 1997, Turner, 1999 has distinguished between ‘higher-level’ repetitive symptoms (including insistence on the maintenance of sameness and circumscribed interests) and ‘lower-level’ repetitive motor actions. Importantly, previous studies of the repetitive tendency has revealed a unitary factor amongst individuals with ASD (South, Ozonoff, & Mcmahon, 2005), with the ‘insistence on sameness’ factor emerging reliably across studies of children with ASD (Leekam, Prior, & Uljarevic, 2011). Thus the cognitive and behavioural symptoms of these characteristics may be different signs of a single underlying tendency. However, the cognitive mechanism underneath these repetitive behaviours observed in ASD individuals, and the extent to which they extend into very high-level decision-making is far from clear at this stage. This study aims to examine whether the ‘desire for sameness’ (which might perhaps be labelled “consistency of behaviour” in some contexts) can in part determine performance on a gambling task, thus demonstrating this tendency at a very high level in the decision-making process.

Turning now to the design of gambling paradigms in social neuroscience, the “executive dysfunction” framework for understanding ASD symptoms tends to suppose that the poor regulation or impaired control mechanisms that lead to repetitive behaviours might be associated with cognitive inflexibility (see Geurts et al., 2009, Hill, 2004, Kenworthy et al., 2008 for review). Gambling paradigms provide the opportunity to investigate response patterns in uncertain situations, and to see the degree to which a tendency towards “sameness” exists. In the classic IOWA gambling test (IGT) studies, Bechara et al. (1994) ask participants to make choices to potential positive and negative outcomes which required them to learn to make advantageous choices based on trial-by-trial feedback as the test progressed. It has been shown that TD individuals are able to identify and then fixate upon the advantageous deck. As noted above, previous lesion and neuroimaging studies have established the link between successful IGT performance and the prefrontal cortex (PFC) region (Bechara et al., 1998, Ernst et al., 2002, Northoff et al., 2006), thus demonstrating the essential role of the PFC region – at least in neurological patients – in risk-taking choices. The IGT has been used with ASD populations before. For instance, Johnson, Yechiam, Murphy, Queller, and Stout (2006) used the IGT to evaluate risk-taking behaviours in adolescents with ASD, and reported no deficits in advantageous deck selection compared with the control group. However, the ASD group made shorter consecutive runs of selecting the advantageous deck than the control group, and showed a constant shift between the four alternative decks. South et al. (2014) used the IGT in adolescents with ASD, and on the other hand, found a significant group × block interaction driven by more frequent and longer runs of the advantageous deck selection over time in the ASD group, compared with the control group. It is not clear at this stage what conclusion can be drawn from these findings. But they do suggest investigating risk-taking behaviours using an item-based approach may be a useful way forward.

Other forms of gambling situation have also been used with people with ASD diagnoses. For instance, in order to investigate if ASD individuals would demonstrate inflexible gambling behaviours to changes of experimental contexts, De Martino, Harrison, Knafo, Bird, and Dolan (2008) designed a task that required both ASD and TD participants to make gambling decisions by comparing a sure and a gamble option with balanced expected value under gain and loss frames. The concept of “expected value” represents, intuitively, the long-run averaged value of repeated gambles by considering both outcome magnitude and its probability in theory. De Martino et al.'s (2008) result showed that adults with ASD demonstrate a reduced “framing effect” (Tversky & Kahneman, 1981). This is a classic cognitive bias where people respond differently depending on how the situation is presented, in this case a tendency to avoid potential risks when positive outcomes are expected, but to seek risks when negative outcomes are expected. Their ASD participants showed similar risk-taking behaviours between the gain and the loss frames. This reduced sensitivity to different frames was interpreted in terms of a failure to integrate contextual information regarding potential rewards and punishments, which could perhaps be viewed as an index for the supposed inflexibility problems in ASD adults.

In order to systematically examine the atypical gambling decisions in ASDs, Weller, Levin, Shiv, and Bechara (2007) developed the Cups task, which involved showing pairs of sure and risky options varying in their expected values to investigate people's choices in risk advantageous (RA), risk disadvantageous (RD), or neutral (equal expected values, EQEV) situations. Compared with the sure option (100% chance), rational gamblers would make more risky decisions in RA situations due to the relatively higher expected values, and make fewer risky decisions in RD situations due to the relatively lower expected values. Weller et al.'s (2007) results showed that patients with lesions to ventromedial prefrontal cortex display an insensitivity to manipulations of expected value by showing more risk-taking decisions to risk-disadvantageous trials (i.e., those where the probability of outcome favours a more conservative approach) than controls in the loss domain. This insensitivity to specific kinds of gambling trial under specific frames suggested that people with structural abnormalities in ventromedial prefrontal structures might take risks in an irrational way (e.g., maintain risk-seeking actions when it is contextually risk-disadvantageous), and is strongly redolent of the original IGT studies of Bechara, Damasio and colleagues. It is not implausible that some people with ASD might perhaps show a similar pattern, since previous studies have reported structural abnormalities in medial prefrontal regions using voxel-based morphometry (Waiter et al., 2004) and using post-mortem PFC neuron numbers (Courchesne et al., 2011) amongst people with ASD. Accordingly, we adopted this general methodology in order to investigate the possibility that the tendency towards sameness or repetitive behaviour is influential in determining gambling choices in ASD individuals, and investigate their gambling patterns under situations varying in expected values (neutral, risk advantageous, risk disadvantageous) under different frames (gain *vs*. loss).

So we developed a gambling test modified from the Cups task (Levin et al., 2007, Weller et al., 2007). However, in order to enable us to measure repetitive tendencies in ASD *in situ*, rather than use a correlation with an external measure as has been typical in the field (see above), we made some critical manipulations to the presentation of the gambling trials. First, each risky versus sure combination (e.g., 50% to win £2 *vs*. 100% to win £1) was presented nine times in each frame. This allowed us to calculate how consistent (or repetitive) the participants were in their responses to identical gamble combinations, and is a measure of risk-taking behaviour beyond the traditional index of e.g., risk rate. Second, the gambling trials under the win and the lose frames were presented separately. This enabled participants to develop a stable “response mode” to potential gains and losses without constant shifts of frames. As a result, this gambling test enabled us to examine the sensitivity to expected values amongst ASD participants by showing atypical repetitive behaviours in risky decision-making to potential gains and losses. Based on previous ASD studies showing rigid response pattern using the IGT, we first hypothesised that adults with ASD would demonstrate a higher repetition of their preferred options in our gambling test. A key aspect of our design is that we measure the tendency towards repetitive behaviour *within* the test, rather than relying on correlations with an external measure. Furthermore, since it has been shown that clinical populations with structural abnormalities in the medial PFC region can make irrational gambling decisions, and also that they can show bizarre and idiosyncratic responding on other forms of executive task (e.g., the Brixton Test; Burgess & Shallice, 1996), and that medial PFC abnormalities are commonly found in ASD populations (e.g., Courchesne et al., 2011, Gilbert et al., 2008, Waiter et al., 2004) we hypothesised that adults with ASD would show atypical (i.e., either irrational or idiosyncratic) risky decision-making choices in response to the manipulations of expected value between the win and the lose frames. Given that the aim of the current study is to introduce a novel way to address the possible inflexible response pattern amongst the ASD population, we further examined if a variable that represents repetitiveness could capture the cardinal features of the repetitive mannerism in adults with ASD compared with using the conventional approach, the risk rate.

## 2 Method

### 2.1 Participants

This study was approved by the UCL Research Ethics Committee (ID number: 3825/001), and informed consent was obtained from all individuals included in the study. We recruited forty-seven typically-developed (TD) participants (28 male) and thirty-three autism spectrum disorder (ASD) participants (22 male) aged between 18 and 70, who were native English speakers with no histories of hearing, visual or motor impairments. All the TD participants were volunteers recruited from the Institute of Cognitive Neuroscience subject database with written consents and ASD participants were invited and screened by licensed clinicians. All the ASD participants had clinical diagnoses, and none of the TD participants reported psychiatric or neurological disorders, or any ASD diagnoses amongst their first-degree relatives. Amongst the 33 ASD participants, 9 were diagnosed with high-functioning autism, 24 were diagnosed with Asperger's syndrome by qualified clinicians according to standard diagnostic criteria. These included the Autism Diagnosis Observation Schedule (ADOS; Lord et al., 2000) criteria for autism spectrum or autism, and/or the Autism Spectrum Quotients (AQ; Baron-Cohen, Wheelwright, Skinner, Martin, & Clubley, 2001), using the recommended cut-off of 32. ADOS scores were available for 28 of the 33 ASD participants. 25 of them met the criteria for an ASD. The three participants whose ADOS scores fell below the cut-off, as well as the five without ADOS score, were not excluded as they provided a reliable written clinical diagnosis and their AQs were all above 32. All participants had full-scale Wechsler Intelligence Quotients (FSIQ) greater than 80 (WAIS-III-UK, Wechsler, 1997, 1999; WASI). The ASD and the TD were matched for age (t (78) = .627, *p* = .532), gender (*χ*^*2*^ (1) = .416, *p* = .640), Verbal IQ (t (78) = .389, *p* = .698), and Performance IQ (t (78) = −.536, *p* = .594) (see Table 1).

**Table 1:** Participant characteristics: mean and (standard deviation).

	ASD group	TD group
n	33	47
Age	35.64 (10.67)	34.21 (9.50)
Gender (M:F)	22:11	28:19
VIQ	115.64 (15.00)	114.43 (12.70)
PIQ	110.82 (14.22)	112.43 (12.45)
ADOS^a^	8.18 (3.41)	
AQ^b^	35.85 (8.85)	

a Autism Diagnostic Observation Schedule; maximum score = 18.

b Autism Spectrum Quotients; cut-off score = 32.

### 2.2 Materials and design

The gambling test consisted of two separate scenarios, or ‘frames’, that required participants to make gambling decisions to potential gains and losses, and each frame contained 144 trials. In each trial of the win and the lose frames, participants were asked to choose between a risky option and a sure option presented side-by-side on the screen. All the options were illustrated by pie charts depicting the probability of winning or losing an amount of imaginary money, indicated by varying amounts of £1 (GBP) coins. The sure options were always illustrated by a pie chart showing a 100% chance to win (or lose) a £1 coin (i.e., no risk at all – the participant knows exactly what the outcome will be). The risky options, on the other hand, were illustrated by pie charts showing 4 levels of probability (20%, 25%, 33%, or 50%) to win (or lose) 4 amounts of money (£2, £3, £4, or £5). Together these probability × money combinations made 16 levels of expected values ranging from a minimum of £0.40 (20%  ×  £2, i.e., the participant has a one in five chance of winning or losing £2) to maximum of £2.50 (50% × £5, i.e., the participant has a 1 in 2 chance of winning £5) amongst the risky options. Compared with the sure options, the risky versus sure combinations could be categorised as: 1) risk advantageous (RA) trials: combinations that favoured risk-taking behaviours with higher expected values in the win frame and lower expected values in the lose frame (e.g., 50% to win £4 *vs*. 100% to win £1 and 25% to lose £2 *vs*. 100% to lose £1); 2) risk disadvantageous (RD) trials: combinations that favoured risk-aversive behaviours with lower expected values in the win frame and higher expected values in the lose frame (e.g., 20% to win £3 *vs*. 100% to win £1 and 33% to lose £5 *vs*. 100% to lose £1); 3) equal expected values (EQEV) trials: combinations that favoured neither risk-taking nor risk-aversive behaviours with identical expected values to the sure options in the win and the lose frames (e.g., 33% to win £3 *vs*. 100% to win £1 and 50% to lose £2 *vs*. 100% to lose £1). The win and the lose frames were each comprised of nine blocks, with 16 trials in each block, where each of the 16 different risky versus sure combinations appeared once in each blocks. The outcomes of all trials were pre-determined instead of reflecting the real probability. For example, a 25% to win £4 versus 100% to win £1 was pre-determined to offer a positive outcome (win £4) as long as a risky decision was registered, and a fixed outcome (win £1) would be offered when a safe decision was made. This pre-determined outcome was the same between all participants. The order of all trials was presented in an identical order across all participants to ensure the same testing experience when comparing between the TD and ASD groups. This was to avoid subtle differences in task format having unpredictable effects in groups of differing ability. This is preferable to e.g., randomising conditions across participants, and makes the procedure then equivalent to e.g., comparing performances on IQ or memory test scores, where task procedures are usually invariant. The positions of the risky and sure options presented on the screen were counterbalanced to avoid any spatial bias in choices.

### 2.3 Procedure

**Fig. 1:**
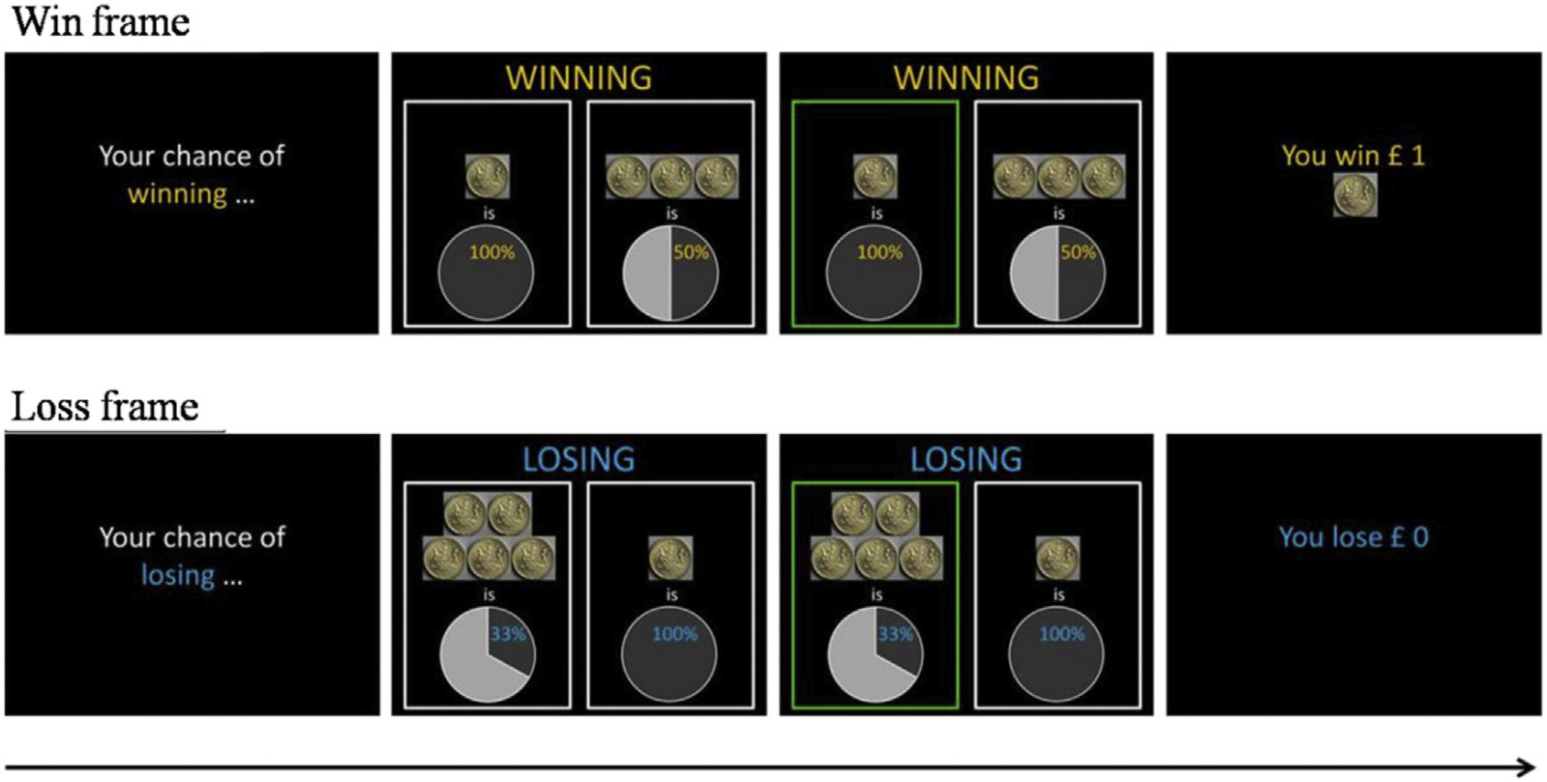
The experimental procedure of the gambling test. All participants administered the win frame first, then the loss frame. In this illustration, participants were required to choose from a sure option to win £1 versus a risky option to have 50% to win £3 in the win frame. In the depicted loss frame, participants were required to choose from a sure option to lose £1 versus a risky option to have 33% to loss £5.

The gambling test was presented on a laptop using MATLAB R2008a (MathWorks) and the script was presented by the Cogent toolbox v1.32. Participants were told that they would be given several chances to win and lose some imaginary money, and were encouraged to end up with as much money as they could (see Fig. 1). To achieve this, all participants performed the win frame first in order to gain some money, and then instructed to perform the lose frame. During each trial, a cue that read ‘Your chance of winning/losing) … ’ would be presented on the screen for 500 msec in the win/lose frame, and two options (a sure option and a risky option) were presented side-by-side on the screen. Participants were told to choose one of the options by pressing an arrow key that pointed in the direction of the option they wanted to choose (i.e., using left or the right arrow keys). Each trial remained displayed on the screen until the participant made a response. When the response was made, the choice option that was selected was highlighted with a green border for 300 msec, and the outcome of that trial was presented on the screen for 700 msec. If participants chose the sure option, the outcome would always be ‘You win/lose £1’ in the win/lose frame. If participants chose the risky option, the outcome would be ‘You win £0/£2/£3/£4/£5’ in the win frame, and ‘You lose £0/£2/£3/£4/£5’ in the lose frame depending on the pre-determined outcomes in different blocks. There were two 10-s breaks at the end of the third and the sixth blocks in the win and the lose frames respectively. The gambling test took each participant approximately 20 min to administer.

### 2.4 Measurements

Three variables were analysed from this gambling test: (1) the risk rate, i.e., the likelihood to make risky decisions; (2) the reaction times, i.e., the time it took participants to make their choices; (3) repetitiveness, i.e., a measurement that represented the degree of consistency in making the same decision (either risk or play it safe) on the gambling trials. The repetitiveness variable, unlike the risk rate, measured the *consistency* of choice when presented the same combination several times within participants. To elaborate on this, a trial depicting ‘a 50% chance to win £2 versus a 100% chance to win £1’ appeared 9 times in each frame. So the frequency in taking a risk when presented with this particular risky versus sure combination ranged from 0 (never took a risk) to 9 (always chose the risky option). A repetitiveness score out of a maximum possible 9 instances is calculated by the following scheme: 0->5, 1->4, 2->3, 3->2, 4->1, 5->1, 6->2, 7->3, 8->4, and 9->5. This ‘V-shape’ transformation weights extreme risk-taking and risk-avoiding behaviours by giving them higher scores. On this basis, making the same decision to one particular combination 4 or 5 out of 9 instances could be considered ‘less repetitive’, whereas choosing the same option under that particular combination 0 or 9 out of 9 instances could be considered ‘more repetitive’. To yield this repetitiveness variable, we first calculated the frequencies in risk-taking for the combinations varying in 16 levels of expected values for the risky options, and then transformed the frequencies according to the ‘V-shape’ weighting formula to yield a repetitiveness score. We summed the repetitiveness score for each participant and linearly transformed this score into a percentile for ease of comprehension. For example, amongst the total of 144 trials in the win frame, if a participant chose the ‘50% chance to win £2’ rather than the ‘a 100% chance to win £1’ in nine out of the total of nine instances, and never chose the risky option on the rest of the 135 trials, the risk rate in the win frame is calculated as 6.25% (9/144), but the repetitiveness in the win frame is calculated as 1.00. In the lose frame, e.g., if a person chooses the ‘33% chance to lose £4’ over the ‘a 100% chance to lose £1’ only once out of the total of nine opportunities, and ‘25% chance to loss £5’ over the ‘a 100% chance to loss £1’ on six out of the total of nine occasions, and never chose the risky option in the other 137 trials, their risk rate in the lose frame would be calculated as 4.86% (7/144). To calculate the repetitiveness, each combination that yielded no risky decision would be transformed to a score of 5, the 1 out of 9 instance combination would be transformed to a score of 4, the 6 out of 9 instance would be transformed to a score of 2, and the summation of scores to the total 16 kinds of combinations would be 76. Next we linearly transformed the score of 76 into a .94 percentile (76 out of a range between maximum 80 and minimum 16), which gave us .94 repetitiveness in the lose frame.

## 3 Results

Experimental variables representing risk rate, repetitiveness, and reaction time were entered into a repeated measures ANOVA with frame (win *vs*. lose), expected values (risk advantageous, equal expected value, risk disadvantageous) as within-subject factors, and group (TD *vs*. ASD) as between-subject factor (see Table 2 for results). For post-hoc examinations, the corrected *p*-value were adjusted in accordance with the numbers of *t*-tests conducted. Therefore the reported results in relation to the effect of frame (win *vs*. lose) was set as *p* = .025, and the effect of expected values (risk advantageous *vs*. risk disadvantageous *vs*. equal expected values) was set as *p* = .017. No Bonferroni corrections were applied for *p*-values when evaluating effects in ANOVA.

**Table 2:** The mean and standard deviation (SD) of the risk rate, the repetitiveness, and the reaction time (msec.) to gambling trials varying in expected values in the win and the loss frames. RA: risk advantageous, EQEV: equal expected value, RD: risk disadvantageous.

Variable	Frame	EV	ASD group		TD group		Sig.
			Mean	SD	Mean	SD	
Risk rate	Win	RA	.65	.33	.76	.25	
		EQEV	.31	.31	.45	.29	a
		RD	.12	.20	.17	.21	
	Lose	RA	.75	.24	.72	.23	
		EQEV	.49	.28	.46	.28	
		RD	.29	.28	.29	.28	
Repetitiveness	Win	RA	.86	.22	.77	.23	
		EQEV	.78	.21	.62	.22	a
		RD	.80	.21	.76	.18	
	Lose	RA	.70	.28	.69	.24	
		EQEV	.58	.29	.60	.25	
		RD	.67	.30	.63	.24	
Reaction time (msec.)	Win	RA	1649.24	493.14	1342.76	512.88	a
		EQEV	1612.35	473.77	1384.20	523.96	a
		RD	1596.59	520.79	1405.56	611.74	
	Lose	RA	1672.57	693.38	1456.32	676.50	
		EQEV	1623.59	597.38	1432.28	557.97	
		RD	1805.01	610.59	1543.17	726.33	

a Independent *t* test showed between group effect *p* < .05.

### 3.1 Risk rate

Repeated measures ANOVA identified a significant main effect of frame (F(1,78) = 7.446, *p* = .008) and of expected value (F(2,156) = 188.051, *p* < .001) across all participants. This indicates that participants took significantly more risks in the lose frame than in the win frame, and the propensity to take risks was in the risk advantageous > equal expected value > risk disadvantageous order (all pair-wise comparisons *p* < .001 using Bonferroni correction). However, there was no significant main effect of group (F(1,78) = .997, *p* = .321). Thus, ASD participants showed no tendency to make either more or fewer risky choices compared with the TD participants. In addition, no significant frame × group interaction (F(1,78) = 3.766, *p* = .056), expected value × group interaction (F(2,156) = .181, *p* = .834), or frame × expected value × group interactions (F(2,156) = 2.065, *p* = .130) were found.

### 3.2 Repetitiveness

**Fig. 2:**
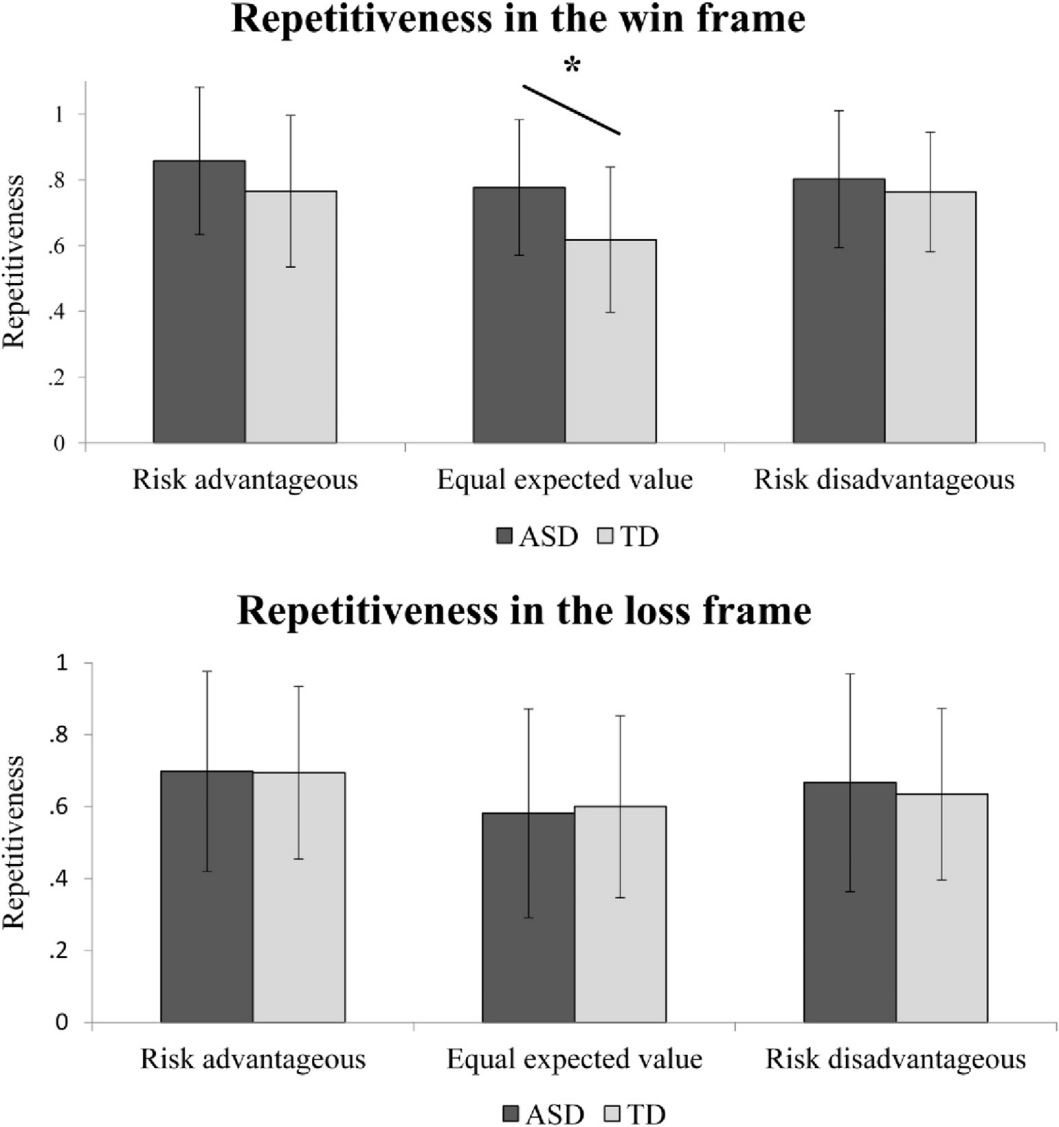
Illustration of the significant frame × expected value × group interaction in repetitiveness. Adults with ASD showed significantly enhanced repetitive choices compared to the TD group only to equal expected value trials in the win frame. Error bars refer to standard deviations.

Across all participants (both ASD and TD), repeated measures ANOVA revealed a significant main effect of frame (F(1,78) = 23.460, *p* < .001) and expected value (F(2,156) = 15.420, *p* < .001), indicating a significantly higher degree of repetitive behaviours in the win frame than in the lose frame, and that the repetitiveness was in the risk advantageous > risk disadvantageous > equal expected value order (all pair-wise comparisons *p* < .05 using Bonferroni correction). There were no significant main effect of group (F(1,78) = 1.817, *p* = .182), no significant frame × group interaction (F(1,78) = 3.527, *p* = .064), and no expected value × group interaction (F(2,156) = .288, *p* = .750). Importantly, however, a significant frame × expected value × group interaction (F(2,156) = 3.201, *p* = .043) was identified (see Fig. 2 for illustration). Given that in post-hoc analysis we conducted six independent tests, which included comparing group differences of the repetitiveness to risk advantageous, risk disadvantageous, equal expected values trials in the win and the lose frames, we used an adjusted *p*-value threshold of .05/6 = .0083. Follow-up analysis confirmed that the ASD group showed significantly higher repetitiveness than the TD group only to equal expected value gambling trials in the win frame (t (78) = 3.262, *p* = .002), but not in any other gambling trials (all *p* > .05). So, in summary, ASD participants only showed significantly higher response consistency in gambling situations that favoured neither risky nor safe options when dealing with potential gains (e.g., a 33% to win £3 *vs*. 100% to win £1).

### 3.3 Reaction time

Repeated measures ANOVA found a significant main effect of expected value (F(2,156) = 5.631, *p* = .004), where the reaction time was significantly longer in risk disadvantageous trials than in equal expected value trials (*p* = .004 using Bonferroni correction) across all participants. There was no significant main effect of frame (F(1,78) = 2.412, *p* = .124), but a significant frame × expected value interaction was identified (F(2,156) = 4.884, *p* = .009). Follow-up analysis showed that the reaction time to risk disadvantageous trials was significantly longer in the lose than in the win frame (t (79) = 2.253, *p* = .027), but the difference was not significant to risk advantageous (t (79) = 1.334, *p* = .186) and equal expected value trials (t (79) = .585, *p* = .560). This indicated that all participants made their decisions significantly slower when facing potential losses than gains only under situations favouring playing it safe (e.g., a 50% chance to lose £4 *vs*. 100% chance to lose £1). Repeated measures ANOVA revealed a significant effect of group (F(1,78) = 4.091, *p* = .047), i.e., ASD participants took significantly longer time to make decisions than TD participants. No significant expected value × group (F(2,156) = .642, *p* = .528) or frame × expected value × group interactions (F(2,156) = 1.494, *p* = .228) were found.

### 3.4 Extreme approaches in risk-taking decisions

We noticed that there were multiple occasions of extreme risk-taking or risk-avoiding behaviours (e.g., never chose the risky option or always took risks throughout the test) in both the ASD and the TD groups. Accordingly we examined if the number of participants who made extreme choice decisions was similar between the groups. We define extreme risk-taking approaches as where an individual either chose the risky option all the time, or chose the safe option all the time, which would lead to the highest level (1.00) and the lowest level (.00) of the repetitiveness variable (i.e., made the same decision to the particular risk *vs*. sure combination all the time) (see Table 3, for results). Fisher's exact test revealed that the difference between groups was not significant when comparing the risk rate variable (risk rate = .00 in the win frame: *χ*^*2*^ (1) = 2.922, *p* = .167, two-tailed; risk rate = 1.00 in the lose frame: *χ*^*2*^ (1) = 1.442, *p* = .412, two-tailed). But when comparing the repetitiveness variable, the ASD group had a significantly higher proportion than the TD group of cases of win frame extreme repetitiveness = 1.00 (*χ*^*2*^ (1) = 6.258, *p* = .0.18, two-tailed), as well as lose frame extreme repetitiveness = 1.00 (*χ*^*2*^ (1) = 5.997, *p* = .026, two-tailed).

**Table 3:** The number of participants showing extreme gambling behaviours.

	Frame	ASD group	TD group	Sig.^a^
Risk rate = 1.00	Win	0	0	
	Loss	1	0	.412
Risk rate = .00	Win	2	0	.167
	Loss	0	0	
Repetitiveness = 1.00	Win	6	1	.018
	Loss	0	0	
Repetitiveness = .00	Win	4	0	.026
	Loss	0	0	

a Fisher's exact test, two-tailed.

### 3.5 Change over time

Inherent in the notion of repetitiveness or consistency is the concept of change over time. So we performed a series of analyses that examined changes in behaviour as the task progressed. This was done by introducing an additional factor of stage to the analyses. The data was partitioned by splitting it up into two different ‘stages’ for each frame, considering block 1 to block 4 as the early stages and block 6 to block 9 as the late stages. Repeated measures ANOVAs with frame (win *vs*. lose), stage (early *vs*. late) as within-subject factors, and group (TD *vs*. ASD) as between-subject factor were conducted. We consider in turn the change over these stages in terms of speed of decision-making (as indicated by reaction time), risk rate, and repetitiveness.

#### 3.5.1 Reaction time

**Fig. 3:**
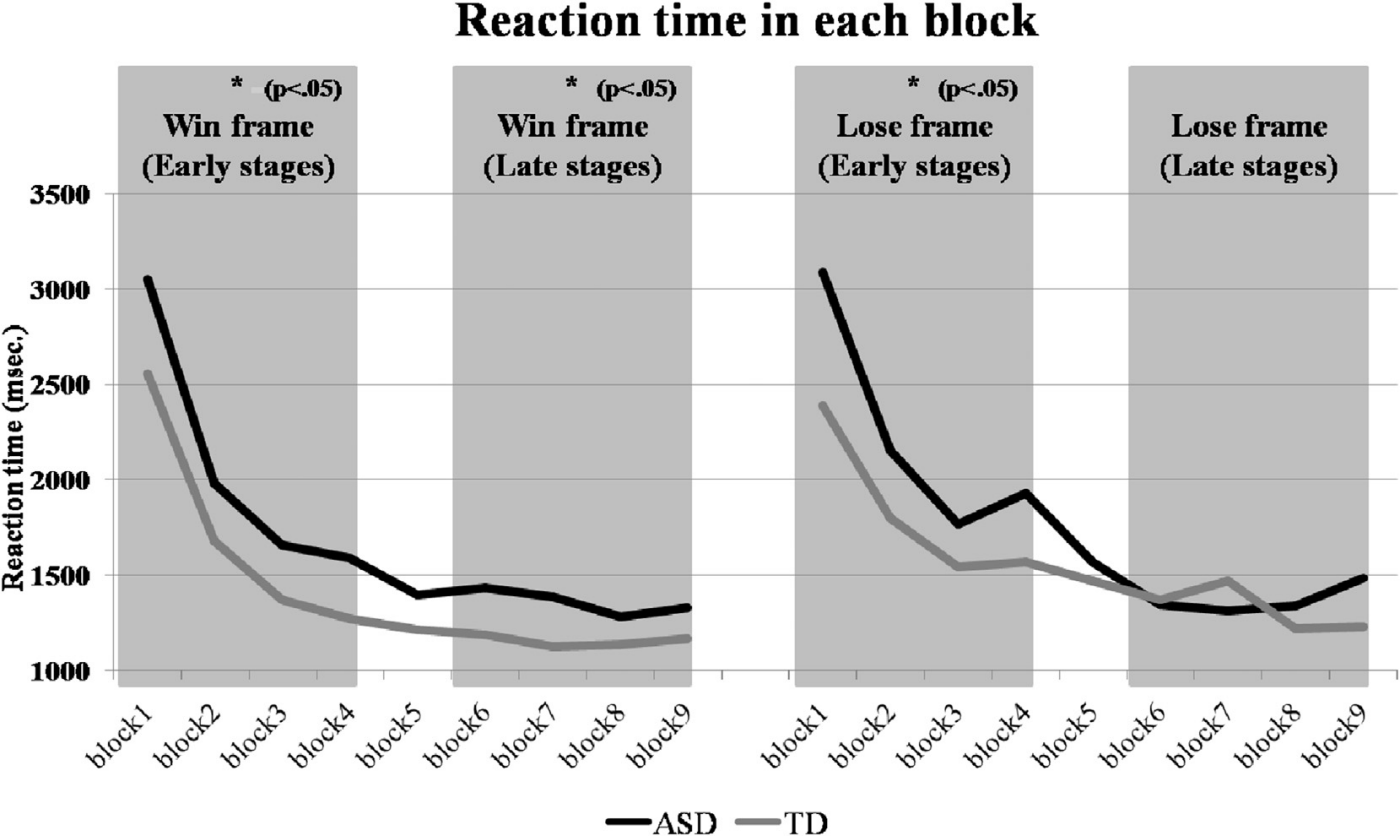
Change in time taken to give responses across the different frames and stages of the experiment. The ASD participants are significantly slower except in the later trials of the lose frame, when they are no different from typically developed controls.

Repeated measures ANOVA of reaction time revealed a significant main effect of stage (F(1,78) = 166.515, *p* < .001), which showed that all participants responded significantly slower in the early stages than in the late stages of each frame. Repeated measures ANOVA identified a significant stage × group interaction (F(1,78) = 6.104, *p* = .016). Post-hoc analysis revealed that the response slowness of the ASD group was only significant in the early stages (t (78) = 2.344, *p* = .022), but the difference was not significant in the late stages (t (78) = 1.273, *p* = .207). Repeated measures ANOVA found a marginally significant group × frame × stage interaction (F(1,78) = 3.788, *p* = .055). Follow-up analysis showed that the ASD participants made their gambling decisions significantly slower than TDs in the early win stages (t (78) = 2.208, *p* = .030), in the late win stages (t (78) = 2.113, *p* = .038), in the early lose stages (t (78) = 2.067, *p* = .042), but the differences were not significant in the late lose stages (t (78) = .366, *p* = .715) (see Fig. 3).

#### 3.5.2 Risk rate

Repeated measures ANOVA of risk rate revealed a significant main effect of stage (F(1,78) = 4.205, *p* = .044), which showed that the tendency to take risks was significantly higher in the early stages than in the late stages of each frame in all participants. Repeated measures ANOVA did not find a significant stage × group interaction (F(1,78) = .948, *p* = .333). No significant group × frame × stage interaction (F(1,78) = 1.122, *p* = .293) was identified.

#### 3.5.3 Repetitiveness

Repeated measures ANOVA of repetitiveness revealed a significant main effect of stage (F(1,78) = 7.690, *p* = .007), which showed that the response consistency of all participants was significantly higher in the late stages than in the early stages of each frame. Repeated measures ANOVA did not find a significant stage × group interaction (F(1,78) = .177, *p* = .675). No significant group × frame × stage interaction (F(1,78) = .781, *p* = .380) was identified.

#### 3.5.4 Relation between repetitiveness and social cognition, IQ, gender, and ASD symptom measures

In order to investigate whether there is a potential link between repetitive behaviours and abilities associated with social cognition in the ASD participants, we administered the Cartoon Faux Pas Test (CFPT) developed by Thiebaut et al. (2016). In the CFPT participants are shown a series of social scenarios depicted in cartoon form, and they are required to say whether the scenario depicts a social faux pas (i.e., and embarrassing social mistake) or not. It is intended as a test of appreciation of social norms and the perspectives of other people. Thiebaut et al. (2016) showed that ASD participants will detect faux pas where they are depicted almost as well as TDs. However they also produce false positives to stimuli that do not show faux pas. Using signal detection analysis, this was shown to be the result of a strategy adopted by the ASD participants under situations of uncertainty. So the key social measure here is the ASD participants' correct hit rates to the non-faux pas stimuli.

A correlation analysis was conducted to investigate the relationships between the repetitiveness in the current study and ability to detect social faux pas. All of the 33 ASD and 47 TD participants in the current study were administered the CFPT. There was a significant positive correlation between repetitiveness in the lose frame and the accuracy of non-faux pas items in ASDs (r = .376, *p* = .031) and TDs (r = .414, *p* = .004) groups. Thus, in both groups, higher response consistency to potential losses was associated with better performance on a sensitive social cognition measure (non-faux pas detection). In the ASD group, higher response consistency to potential losses was also associated with gender (r = −.35, *p* = .041), Verbal IQ (r = .47, *p* = .006), Performance IQ (r = .54, *p* = .001), and AQ scores (r = .41, *p* = .02). As regards the association with gender, male ASD participants demonstrated significantly higher repetitiveness compared to female ASD participants in the lose frame. More specifically, when analysing the overall repetitiveness, putting frame (win *vs*. lose) as a within subject variable, and gender and group as between subject variables, a significant frame × group × gender three-way interaction was revealed (F(1,76) = 11.353, *p* = .001). Follow-up analysis identified that the gender effect was only significant in the lose frame (t (31) = 2.133, *p* = .041) in the ASD group, where the ASD males showed significant higher repetitiveness (mean = .70) than the ASD females (mean = .48). No significant gender effect was found in either frame in the TD group, and none of all the other performance variables from either the gambling test or the Faux pas test showed significant gender effects (all *p* > .05).

Turning now to relationships with symptoms as measured by the ADOS, the ADOS repetitive symptom score was negatively correlated with reaction time in the lose frame, which indicates that the ASD participants who showed higher repetitive behaviours in everyday life tended to make faster gambling decisions when faced with potential losses on our gambling task. Furthermore, a significant positive correlation was identified between AQ score and the repetitiveness in the lose frame, indicating that ASD individuals with more severe autistic traits tended to demonstrate higher repetitiveness in their choices to potential losses.

## 4 Discussion

The aim of the study was to investigate the risk-taking behaviours in ASD, a clinical population characterised by mannerisms that are purportedly associated with a desire for sameness. In terms of overall risk-taking, the ASD participants did not differ from the TD controls: Both groups tended to “gamble” more in the lose than the win frames. This is consistent with the classic “framing effect” (Tversky & Kahneman, 1981) with a cognitive bias towards avoiding potential losses than making potential gains. The significant main effect of expected value in the risk advantageous > equal expected value > risk disadvantageous order indicates that all participants make risky decisions in a rational way. These results therefore demonstrate that the gambling test devised for this study is in general able to provide sensible measurements of gambling choices in our participants.

This does not mean that the performance of the ASD participants was the same as the TD participants, however. Although the ASD participants showed typical response patterns to the framing (win/lose) manipulation, they made their gambling decisions significantly slower than the TD group. This is consistent with a general slowness of responding often observed in ASD studies (Ozonoff, Strayer, Mcmahon, & Filloux, 1994). Possible explanations for this phenomenon are not well-formed. But in the case of the current experiment at least, they might be related to demands upon cognitive flexibility or psychomotor speed (Goldstein et al., 2001), since at a group level, ASD individuals can show executive difficulties with organising information and monitoring on-going events at the conceptual rather than perceptual level (Hill, 2004, Ozonoff et al., 1994). However, there is another possibility related to the “gateway hypothesis” of rostral prefrontal cortex function (Burgess, Dumontheil, & Gilbert, 2007). The gateway hypothesis suggests that rostral prefrontal cortex (principally area 10) supports a cognitive system that facilitates novel degrees of attenuation of attending between either sensory stimuli (known as “stimulus-oriented attending”), or to internally-generated thoughts (known as “stimulus-independent attending”). In everyday language, the idea is that rostral PFC structures allow to you either concentrate, to an unusual degree, on external stimuli to the exclusion of “inner mental chatter”, or alternatively to concentrate on your own thoughts and “shut out the external world”, should you need to. This hypothesis has been tested directly several times by Burgess's research group and also by others (e.g., Burgess et al., 2007, Henseler et al., 2011). One of the predictions of this hypothesis is that there should a relationship between response speed and rostral PFC activation across a wide range of tasks. This prediction was supported empirically by a meta-analysis of neuroimaging studies by Gilbert, Spengler, Simons, Frith, Burgess, (2006), and by direct fMRI experimentation by Gilbert, Simons, Frith, Burgess, (2006). This region of rostral PFC that was found to be associated with relatively faster reaction times across tasks was a medial region that is anatomically very close (but anterior to) to the medial PFC region often associated with mentalizing (for discussion see Gilbert, Dumontheil, Simons, Frith, & Burgess, 2007). Furthermore, this research group has demonstrated that these PFC regions show unusual patterns of functional specialisation in a sample of ASD adults very similar to that tested here (Gilbert et al., 2008, Gilbert et al., 2009). So it may be plausible that perturbations in the rostral PFC “attentional gateway” might lead to slowed reaction times across many tasks in ASD participants.

Clearly, this possibility needs to be tested further. But whatever the source of the slowness in decision-making observed here, this experiment presents an important finding to be considered in its interpretation. We found that a determinant of the size of the reaction time group effect was when the ASD participants had made the decision. The ASD participants were not slower to make their choices in the later stages of the lose frame, whereas they were at other times. This finding may well hold a key to knowing why the ASD participants were mostly slower. Indeed, it may be related to the development and subsequent practice of strategic preferences. In other words, if a person decides upon a particular strategy and applies it consistently, one might expect a practice effect to occur in this person that is larger than someone who is more variable in their choices. On this account, the lack of stage effect in the win frame RTs might be because the TDs tend to stay with their strategy when winning. However, this is only one possibility; the issue remains to be resolved.

Next, the analysis of the variable we calculated specifically to capture the consistency of responding to gambling trials, repetitiveness, identified a significant main effect of frame, where all participants responded more repetitively in the win frame than in the lose frame. This corresponds well with the ‘win-stay, lose-shift’ principle in gambling paradigms (e.g., Nowak and Sigmund, 1993, Robbins, 1952), where people tend to maintain strategies when receiving rewards as a positive reinforcement, and tend to change their response strategies when receiving punishment, presumably because punishment is taken as a signal to change. The significant main effect of expected value in the risk advantageous > risk disadvantageous > equal expected value order suggests that all participants tended to make decisions more consistently when it favoured risk-taking or risk-avoiding actions, whereas decisions made under neutral situations were relatively variable. Critically however, we identified a significant three-way interaction (frame × expected value × group) interaction showing that the ASD adults were more consistent in their gambling choices when facing equal expected value gambling trials in the win frame, compared to TD individuals. Prima facie this is in agreement with the second hypothesis that ASD participants, a population diagnosed with repetitive mannerisms, might demonstrate a higher degree of response consistency when making decisions to potential gains based on the observed ‘win-stay, lose-shift’ strategy here. But in fact the ASD group responded significantly *more* repetitively than the TD group in the equal expected value gambling trials, which is the situation showing the *lowest* repetitiveness in general. Thus consistency of responding was not increased in response to the manipulations that affect the risk decision-making demands of the task, but rather in the absence of them. Why should this be the case? There are many possible explanations. We will consider just two here. The first is related to the finding of White et al. (2009) that adults with high-functioning autism may show greatest atypicality of behaviour on “open-ended” tasks, i.e., those where a particular way of behaving is not tightly signalled or constrained by the test parameters. Choices in the equal expected value frame were more open-ended in this way, in that there was nothing about the stimuli that would strongly suggest one choice over another. They were of equal valence. So this open-endedness may have set a context where the ASD participants were free to stay with past choices, whereas the TD controls were free to make an exploratory or novel choice. This suggests a second possible interpretation, which is that this result may be related to differences between the groups in exploration versus exploitation decision-making strategies. Exploration refers to a phase in decision-making where a participant will try out new approaches. It is inherently risky since some new ways will by definition not turn out to be optimal. But it also may lead ultimately to a better way of doing the task. Exploitation refers to the phase where one has decided upon a strategy and is using (or “exploiting”) it. Daw, O'Doherty, Dayan, Seymour, and Dolan (2006) showed, using gambling tasks, that human participants' behaviour tends to follow a predictable pattern when faced with an exploit/explore dilemma, and they identified the frontopolar cortex and intraparietal sulcus as preferentially active during exploratory decisions. Adult high-functioning autism populations have been shown to display atypical patterns of activation in frontopolar cortex during performance of an executive function task that required switches between stimulus oriented and stimulus-independent attending (Gilbert, Simons et al., 2006, Gilbert, Spengler, Simons, Frith et al., 2006, Gilbert, Spengler, Simons, Steele et al., 2006). So on this account, the consistency of response choices in the equal expected value trials shown here may reflect a tendency towards an exploit rather than an explore decision preference. Further, more speculative possible causes of group differences recognise that most psychometric testing situations are social situations: it might be that the TD controls are influenced by the presence of the experimenter (or being observed by the experimenter) in a way that the ASD participants are not (or vice-versa). Similarly, perhaps the gambling behaviours of the two groups might differ if the recipient of the reward was not the participant. But these are just preliminary hypotheses, and they invite further investigation, especially in relation to the symptoms of repetitive behaviour/restricted interests more generally.

Although the frame × group interaction in repetitiveness was not significant, the ASD participants showed significantly higher repetitiveness only in the win frame and not in the lose frame. Furthermore, we noticed that the ASD participants seemed to more frequently implement an extreme decision-making strategy by showing more cases of extreme repetitiveness = 1.00. We extended this repetitiveness analysis and found that there were a significantly higher proportion of ASD participants who chose the same option every time regardless of the manipulation of expected values in both the win and the lose frames. When looking at the propensity to take risks (i.e., risk rate), of those ASD participants who had the highest repetitiveness of 1.00, only four kinds of risk rate were made, which were .00, .375, 0625, and 1.00. Besides those extreme risk-taking and risk-avoiding strategies (i.e., risk rate = .00 or 1.00), some ASD participants made risky decisions only to RA trials all the way through the win/lose frames (54 out of the total 144 trials), and some took chances to RA and EQEV trials all through the win/lose frames (90 out of the total 144 trials).

There are two implications to this extreme risk-taking performance amongst ASD participants. First, it seems rational that some ASD participants followed the expected value and “played it safe” under the situations favouring risk-avoidance. Nevertheless, those ASD participants maintained their unique strategy all the way throughout the win/lose frames. On the other hand, it appears perhaps that the TD gamblers would try to optimise their decision-making strategies on a more trial-by-trial basis, and occasionally make irrational gambling choices if on “a winning streak”. In the classic ‘exploration – exploitation’ dilemma, gamblers try to optimise decisions on the basis of accumulated experience, the richest option, and the learning process from choosing less familiar option with bigger potential (Cohen et al., 2007, Daw et al., 2006). A significantly higher proportion of ASD participants would exploit their preferred strategies and ignore any contextual changes. Second, analysis at an individual rather than a group level revealed a higher proportion of ASD participants who show enhanced repetitive mannerism in both frames. This is consistent with the multiple case series approach proposed in Towgood, Meuwese, Gilbert, Turner, and Burgess (2009). Towgood et al. showed that it is possible to find an “averaging artefact” in data from ASD participants: if one considers as one group a set of individuals who actually show heterogeneous patterns of performance, the averaged results can be misleading. Thus these data are in agreement with Towgood et al. (2009) in suggesting a case-by-case approach to analysis be used in studies investigating ASD cognitive atypicalities, and not rely only upon data averaged across the samples.

Turning now to comparison with findings from other groups, the current results differ somewhat from those reported by De Martino et al. (2008). In that study, a significantly smaller framing effect was observed in the ASD group compared with the control group, suggesting insensitivity to contextual changes amongst ASD individuals. However, in the current study, no significant frame × group interaction was identified. It is important to note that there were several differences in the formats of the two gambling paradigms, which might contribute to this incongruence in framing effect. First, in De Martino et al. (2008), the gain and the loss trials were mixed and ordered in a pseudorandom way, whereas here the gambling test here used block design to separate the two frames. It is possible that the presentation of gain versus loss frames in either a mixed or separate way may make fundamental differences to the strategies implemented by participants. For example, as ASDs are diagnosed to have enhanced repetitive mannerisms, intuitively one might suppose that ASD individuals would be more vulnerable to frequent switches between frames. In other words, frequent switches of frames might make the two frames indistinguishable to ASD individuals, and therefore led to the attenuated framing effect compared with the control participants. Second, each gambling trial showing a sure and a gamble option in De Martino et al. (2008) was equal in terms of expected values. However in this study there was variation in expected value (e.g., risk advantageous and risk disadvantageous) in the current gambling paradigm. When examining the effect using trials with equal expected values, analysis of the risk rate showed no significant group × frame interaction. But a strong group × frame interaction effect was revealed when analysing the repetitiveness. The ASD participants demonstrated significantly enhanced repetitive behaviours compared to the TD participants specifically to potential gains, but were more willing to explore different possibilities than the TD group when facing potential losses. This finding suggests a possible domain-specific enhanced rigidity of responding in ASD, or another perspective on the ‘framing effect’ demonstrated by response pattern, differing from that using risk rate. Third, in De Martino et al. (2008), four different starting amounts of initial money was offered to participants first, and participants were then required to choose between a sure and a gamble option. The gamble options depicted varying proportions on a pie chart to either ‘keep all’ or ‘lose all’, compared with a sure option with equal expected value (see Fig. 1 in De Martino et al., 2008). Here we did not offer participants any initial money, and each gamble option was compared to a definite win/loss of £1, and both options provided participants the information (e.g., the amount of money and the probability to win/lose it) to calculate the expected values if they wished to (see Fig. 1). It is possible that the sure options in De Martino et al. (2008) were more straightforward to understand, which might enhance the framing effect. To elaborate on this, if ‘keep £20’ and ‘lose £30’ were easier option to understand, compared with the gamble options, participants might tend to choose the more straightforward option in the gain frame to secure the amount of money being shown. In the lose frame, the ‘lose £30’ is a more straightforward option, yet it is a reluctant option to choose when choosing a sure loss. At this decision-making moment, the gamble option might provide participants with a possible escape route to avoid this conundrum. In the gambling paradigm here, we provided straightforward information for participants to consider the expected values when pondering those risky options. Participants might make risky decisions in a more rational way in accordance to their own response strategies. Together, these differences in format between the two gambling paradigms in the two studies might explain this apparent disparity in results related to the framing effect.

In terms of the relation between the tendency towards consistency of responding and measures of social cognition, we examined the performance on the gambling task alongside that on a test of social cognition (detecting embarrassing situations) using the test developed by Thiebaut et al. (2016). In that study, there was general agreement between ASD and TD participants in terms of the “difficulty” of individual test items, when the scenarios did actually depict a ‘faux pas’. In other words, the test items where the TDs often failed to detect the faux pas were also failed by the ASDs, and the ones they got right tended also to be performed well by the ASD group. However, for “non-faux pas” items (i.e., cartoon which might have depicted a faux pas but didn't, this agreement in difficulty did not exist. Thiebaut et al. (2016) suggested that the ToM-related problems found in the ASD sample might not be simply reflect a deficit in social cognition (since they could detect faux pas when they were actually present), but might also relate to the compensatory use of “social schemas” or the other forms of method of constraining response options when one is not sure how one should respond (lO'Hearn, Asato, Ordaz, & Luna, 2008; White, 2013). One such path might be where the ASD participant thinks that are poor at detecting embarrassing behaviours or situations so “over-compensates” when they are uncertain if one has been committed. Hence the increased number of false positives on the CFPT. In our study, we found a significant positive correlation between repetitiveness in the lose frame and the accuracy of non-faux pas items in ASD and TD groups. Thus, higher response consistency to potential losses was associated with better performance on the non-faux pas stimuli items, which are the best indicator of social perception ability. Prima facie, this suggests a relation between gambling behaviour and social cognition which reflects the history of the development of the Iowa Gambling Task, which (as outlined in the Introduction) initially emerged out of studies of neurological patients like EVR who showed social cognition changes likened to an “acquired sociopathy”. However, it is important to note that the relationship between these two variables (higher consistency of choices and performance on the faux pas test) was no stronger in the ASD group than the TD group. In other words, this association cannot be considered a “sign of autism”, even if poor performance on either or both of the individual measures (lose frame repetitiveness; poor detection of social faux pas) might be. Indeed, if anything perhaps the association between the variables was actually slightly lower in the ASD group, which is probably not what would be expected if the underlying processes shared a substrate and there was larger variance in the ASD group. These results therefore possibly mirror the wider debate concerning the relation between social cognition problems and tendency towards repetitive behaviours and interests, where the balance of opinion currently seems to be that they are potentially fractionable (e.g., Happé and Ronald, 2008, Kuenssberg et al., 2011). These results in turn therefore suggest one consideration that might be carried forward into the study of gambling behaviours in neurological patients, in the spirit of inter-disciplinary crosstalk that Rumiati and Humphreys (2015) highlighted.

A further question that may arise when those familiar with the neuropsychological literature consider an unusual degree of repetition of choices is whether this may constitute perseveration. If we make the assumption that faster decision times reflect the operation of a distinct strategy or approach (i.e., one decides how to respond in advance), then one interpretation of the negative correlation between ADOS repetitive symptom scores and reaction time in the lose frame might be that those who show increased repetitive behaviour symptoms have decided in advance what they want to do, and invariably follow that strategy. Possibly, neither our understanding of perseverative phenomena in neurological patients nor repetitive behaviours in ASD participants at an information processing level is currently advanced enough to entirely settle this debate. However, if we consider the different forms of perseveration that are often quoted (e.g., Sandson & Albert, 1984), this casts doubt on whether the ASD repetitive behaviour see here could be easily classified as perseveration. For instance, three forms of perseveration are: stuck-in-set perseveration (which is the maintenance of a current category or mental framework beyond the point it would be appropriate); recurrent perseveration (repetition of a previous response to a subsequent stimulus, but where the intention is not to do so); and continuous perseveration (the continuous repetition or extending of a behaviour to a degree that is inappropriate). In the case of stuck-in-set and continuous perseveration, the definitional requirement that the behaviour is inappropriate perhaps excludes them as a possible label to be applied to our ASD participants' choice histories: it is hard to see in what way the ASD choices can be thought of as “inappropriate”. It did not necessarily result in disadvantage, and it was not forbidden by the rules or purpose of the exercise. The decisions were idiosyncratic rather than inappropriate. For recurrent perseveration, most theorists require that the behaviour not be intended. We have no evidence here that the ASD participants did not intend to make their decisions as they did, and given that they were consistent and rational, have no reason to suppose so. Overall, perhaps a more obvious link with decision-making changes in neurological patients might be with the idiosyncratic choices and strategies adopted by some patients with frontal lobe damage in rule detection tasks (e.g., Burgess & Shallice, 1996). But there is currently too little evidence to know if these idiosyncratic choices are made consistently in neurological cases, as the ASD participants did here. In this way, the patterns of ASD participants present an explanatory challenge to those studying the neuropsychology and cognitive neuroscience of decision-making.

This study does however suggest some correlates of this tendency towards consistency of responding (i.e., repetitiveness) which suggest that they may be clinically noteworthy. In both TD and ASD groups, higher response consistency when faced with potential losses was associated with better performance on a sensitive social cognition measure (non-faux pas detection). Moreover, the male ASD participants showed a higher degree of this consistency than the female ASD participants, and across all the ASD participants increased consistency of choice was correlated with verbal and performance IQ scores as well as AQ scores: ASD participants with higher AQ scores (i.e., indicating a higher presence of autism-like symptoms) were more repetitive in their choices. Interestingly, faster gambling decisions when faced with potential losses was associated with increased symptoms of repetitive behaviours on the ADOS. This may suggest perhaps that the highly consistent (or repetitive) individuals are enacting a pre-determined choice strategy (i.e., exploitation) rather than considering the possibility of a new approach (i.e., exploration). However, it may also be a result of quicker strategy development, or better recollection of previous decisions, which would be consistent with the association with better psychometric test scores. It remains a possibility that speed of decision-making and degree of repetitiveness have different causes. One potential link between them may however be related to a tendency towards avoidance of novelty. This putative explanation remains to be tested directly.

In conclusion, the results here suggest that considering the consistency of responses in gambling-type paradigms, or indeed, perhaps other decision-making type paradigms, may yield results that only considering overall choices may not. We have shown that at the group level, analysis of the repetitiveness (or to use a perhaps less pejorative term, consistency of choice) highlighted that the ASD participants made more similar decisions to equal expected value gambling trials in the win frame than TDs. On the individual level, there were significantly more ASD participants who used the same choice strategy throughout the entire win and/or lose frames without, apparently, exploring alternatives. Compared with previous studies that have used risk rate (e.g., De Martino et al., 2008) to demonstrate insensitivity to contextual changes and length of advantageous deck selection in the IGT (e.g., Johnson et al., 2006, South et al., 2014) as an indicator of cognitive inflexibility, the repetitiveness variable used here provides an item-based approach to elucidate the cardinal nature of the confined interests in ASDs, and allows identifying the atypical response strategy in the individual level. Overall, this finding suggests that consistency of choices could be used as a more representative variable in capturing the characteristics of ASD individuals, compared with risk rate, which depicts risk-taking behaviours from a more macro perspective. It also highlights the issue of considering how the unique behavioural propensities of people with ASD might challenge simple accounts of the construct validity of decision-making tasks, and the need therefore to consider methods of analysis that can take them into account. In particular, there is a need to understand how differences in the precise format and presentation of gambling-type tasks can bring out differences in strategies and propensities in populations that may explain some apparently conflicting results (e.g., between Johnson et al., 2006 and South et al., 2014; see Introduction). It seems likely that format issues that may change the development of strategies over time, or the degree to which participants are making informed decisions about the risks they are taking, rather than having to discover them for themselves, as well as their implicit understanding of the motives and purpose of the experiment, the likely reward contingencies, and of their own personal attitudes towards risk, which will all impact upon the precise pattern of choices demonstrated, in addition to the tendency towards repetitiveness. Since the former kinds of processes that underpin these reactions to format changes will likely also be shared to some degree with tasks measuring novel problem-solving or social cognition, then gambling tasks may provide an interesting intersection between these disciplines, providing data that can speak to both.
